# CoupTFI Interacts with Retinoic Acid Signaling during Cortical Development

**DOI:** 10.1371/journal.pone.0058219

**Published:** 2013-03-05

**Authors:** Susan J. Harrison-Uy, Julie A. Siegenthaler, Andrea Faedo, John L. R. Rubenstein, Samuel J. Pleasure

**Affiliations:** 1 Department of Neurology, University of California San Francisco, San Francisco, California, United States of America; 2 Department of Psychiatry, University of California San Francisco, San Francisco, California, United States of America; 3 Programs in Neuroscience and Developmental Biology, Eli and Edythe Broad Center of Regeneration Medicine and Stem Cell Research, University of California San Francisco, San Francisco, California, United States of America; University of Fukui, Japan

## Abstract

We examined the role of the orphan nuclear hormone receptor CoupTFI in mediating cortical development downstream of meningeal retinoic acid signaling. CoupTFI is a regulator of cortical development known to collaborate with retinoic acid (RA) signaling in other systems. To examine the interaction of CoupTFI and cortical RA signaling we utilized Foxc1-mutant mice in which defects in meningeal development lead to alterations in cortical development due to a reduction of RA signaling. By analyzing CoupTFI^−/−^;Foxc1^H/L^ double mutant mice we provide evidence that CoupTFI is required for RA rescue of the ventricular zone and the neurogenic phenotypes in Foxc1-mutants. We also found that overexpression of CoupTFI in Foxc1-mutants is sufficient to rescue the Foxc1-mutant cortical phenotype in part. These results suggest that CoupTFI collaborates with RA signaling to regulate both cortical ventricular zone progenitor cell behavior and cortical neurogenesis.

## Introduction

During cortical development neurons are produced in a tightly regulated manner from progenitor cells residing adjacent to the lateral ventricles. In the developing cortex bipolar radial glia are the primary neurogenic stem cells. These bipolar cells span the cortical wall with cell bodies close to the ventricular surface and primary cilia projecting apically into the ventricle, receiving signals that promote radial glia proliferation [1], [2]. Radial glia also have basal processes projecting to the pial surface where the radial glia endfeet interact with the pial extracellular matrix [3], [4]. Our previous studies suggest that meningeally derived retinoic acid (RA) promotes neurogenesis of radial glia by interacting with the radial glia basal process [5]. However, radial glia also receive signaling cues from neighboring progenitor cells and differentiated neurons [6], [7], [8], [9]. Thus, numerous extrinsic signals from the surrounding environment during development impact the behavior of radial glia, collaboratively regulating their proliferative capacity and developmental maturation.

Foxc1 is a transcription factor expressed by cells in the meninges but not by neural cells in the cortex [10]. Disruption of Foxc1 expression results in a failure of meningeal cells to appropriately migrate and surround the developing forebrain [5], [10]. In addition to the defects in meningeal development Foxc1 mutants have major cortical developmental abnormalities, with elongation of the cortical neuroepithelium, changes in the composition of the progenitor cell population, and decreased neurogenesis, all suggesting a failure to transition from symmetric proliferative divisions to asymmetric neurogenic divisions [5]. Several lines of evidence support a role for meningeal derived RA in controlling cortical neurogenesis [5]. RA is a potent neurogenic factor that regulates areal patterning and neuronal differentiation during development [11]. Disruption of RA signaling, via loss of retinoic acid receptor (RAR) or retinoid X receptor (RXR) family members, results in severe developmental abnormalities across multiple organ systems [12]. Expression of a dominant negative RAR construct in the developing forebrain results in altered proliferation of progenitor cells and increased cell death [13]. However, how RA regulates the process of neuroepithelial progenitor division is still very much unclear.

CoupTFI is a nuclear orphan receptor that can function as a repressor or activator of gene expression via complex, context dependent interactions with other nuclear orphan receptors and transcription factors [14], [15]. In the developing forebrain CoupTFI is expressed in a high-ventral to low-dorsal and high-caudal to low-rostral gradient, initially in progenitor cells (including radial glia cells) and during later development also in neurons [16]. CoupTFI mutant mice have altered areal patterning of the cerebral cortex [17], [18], [19] and CoupTFI also regulates neuronal differentiation and neuron cell type specification [17], [19], [20].

Several lines of evidence suggest that the transcription factor CoupTFI may coordinate the response of cortical progenitor cells to RA. First, CoupTFI binds to RXR and forms DNA heterodimers with both RAR and RXR family members [14], [15]. CoupTFI also interacts with the ligand-binding domain of RAR and RXR family members, resulting in transrepression of retinoid family members [14], [15]. Second, CoupTFI binds to similar DNA binding sites as RAR and RXR, and may function as a negative regulator of retinoid function [14], [15]. Third, CoupTFI-knockout and CoupTFI-overexpressing mice exhibit cortical phenotypes consistent with roles for CoupTFI in regulating neuronal differentiation [17]. Finally, transgenic mice overexpressing CoupTFI in cortical progenitor cells exhibit elevated levels of endogenous RA signaling (See Faedo et al., in their Supplementary Material: Figure S6E–F′ of [17]).

In this study we examined the relationship between CoupTFI and RA signaling in the developing cortex using Foxc1-mutant mice. We found that CoupTFI is required for RA mediated rescue of Foxc1-mutants and that overexpression of CoupTFI in cortical progenitor cells partially rescues the Foxc1-mutant phenotype. Our studies suggest that CoupTFI interacts with RA signaling to regulate cortical progenitor cells.

## Materials and Methods

### Animals

Embryos were obtained from matings of Foxc1^hith^, Foxc1^lacZ^, D6-CoupTFI, CoupTFI-null lines and genotyped as previously described [5], [17], [21], [22]. Noon on the day of vaginal plug was designated as embryonic day 0.5 (E0.5). For RA treatment, pregnant mice were fed all-trans (at) RA-enriched food (250mg atRA/kg food; Harlan Teklab Custom Diets) ab libitum from E10. Mice consumed on average 20–30 mg atRA/kg body weight. Embryos were collected via cesarean section, fixed in 4% paraformaldehyde for 5 hours (E12.5) or overnight (E14.5), processed through a sucrose gradient, and embedded in OCT for cryosectioning. Embryos were cryosectioned at 12 µm for immunohistochemistry or 20 µm for *in situ* hybridization. All animal protocols were approved by the University of California, San Francisco Institutional Animal Care and Use Committee.

### Immunohistochemistry

Immunohistochemistry and cresyl violet staining was performed as previously described [10], [23] using the following antibodies: mouse anti-BrdU (1∶75, BD Biosciences); rat anti-Ctip2 (1∶500, Abcam); rabbit anti-Ki67 (1∶400, Thermo Scientific); rabbit anti-Pax6 (1∶500, Abcam); rabbit anti-Tbr1 (1∶1000, Abcam); rabbit anti-Tbr2 (1∶1000, Abcam). Primary antibodies were detected using secondary antibodies conjugated to Alexa fluorophores (Invitrogen). Six serial 12 µm sections were stained for each antigen in the following order: CoupTFI double mutant experiment: CV, Tbr2, Tbr1/Ctip2, Pax6. D6-CoupTFI E14.5 experiment: CV, X, Tbr2, X, Pax6, BrdU/Ki67, Tbr1/Ctip2 (X: indicates a skipped slide). Stained sections were visualized on a Nikon fluorescent microscope and captured with a digital CCD-cooled camera and QCapture Pro software (QImaging Surrey). Composite images were prepared in Adobe Photoshop CS4 and Adobe Illustrator CS4. Contrast, color and brightness were adjusted in Adobe Photoshop CS4.

### *In Situ* Hybridization

RNA *in situ* hybridization was performed as previously described [24]. Probes used were CoupTFI and CoupTFII (gifts of M.J. Tsai).

### Image Analysis, Quantification, and Statistical Analysis

**Figure 1:**
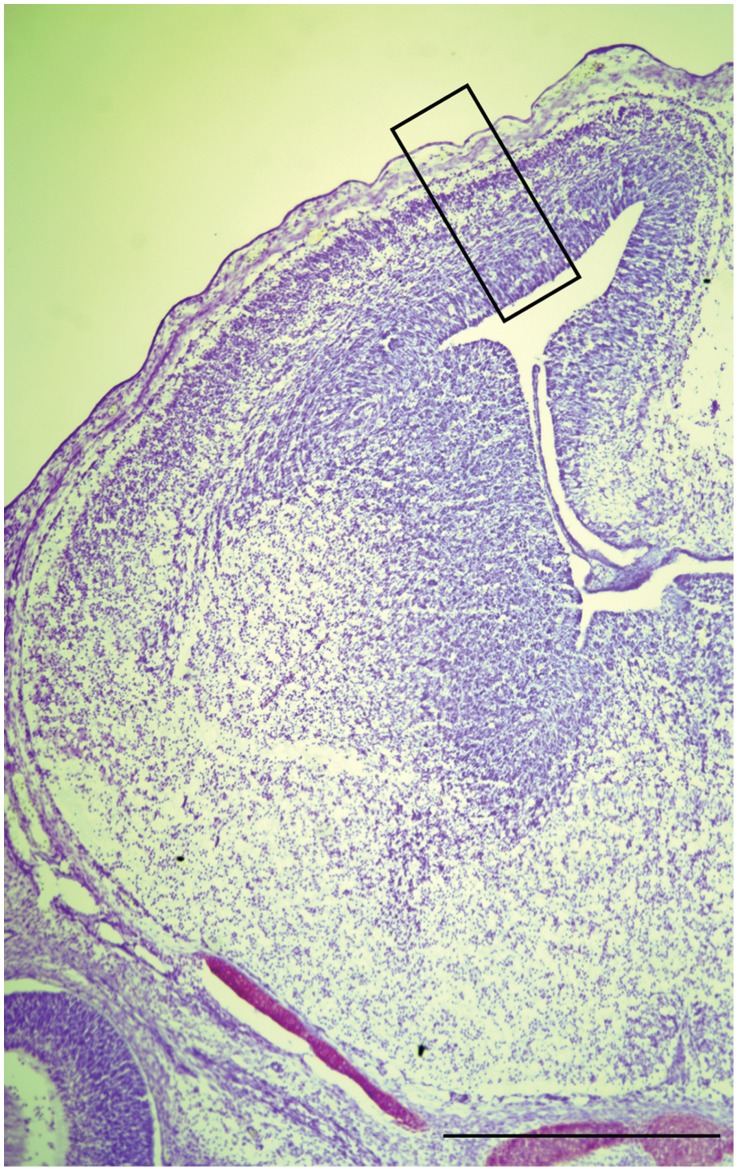
Sampling Window Used For Cell Counts. The location of the 150 µm sampling window in the medial-lateral dimension of the cortex spanning from the ventricular to pial surface of the dorsal cortex is identified by the black box in this coronal forebrain section of an E14.5. Scale bar = 500 µm.

Sections were histologically matched for rostral-caudal level between genotype. The length of the dorsal forebrain consisted of measuring the length of the ventricular surface from the cortico-striatial boundary to the boundary of the cortical hem in sections from a similar rostral/caudal position in ImageJ. Three matched sections per animal were quantified and the mean of the three sections per animal was compared between genotypes. Cell counts were performed and quantified using a cell counting program, as previously described [25]. For all cell counts a matched 150 µm window in the medial-lateral dimension spanning from the ventricular to pial surface of the dorsal cortex from three sections per animal were counted, and the mean of the three sections per animal was compared between genotypes. A minimum of 3 brains per genotype was analyzed from a minimum of two independent litters. The 150 µm counting window was chosen based upon the following criteria. The medial wall of the cerebral cortex, the hippocampal primordium, has distinct molecular properties [26] and in Foxc1-mutants the lateral meninges is intact [5]. In D6-CoupTFI animals, the D6-promoter drives expression of CoupTFI in the medial and dorsal cortex [17], [27]. Therefore, we chose a 150 µm region in the dorsal cortex (as illustrated in Figure 1) to examine the role of these signaling pathways in cortical progenitor cells. We also calculated the total area of the 150 µm counting window, and the proportion of DAPI^+^ cells in the total area of the counting region. The Q-fraction was calculated as the number of BrdU^+^Ki67^−^ cells divided by the total number of BrdU^+^ cells. An observer blind to the genotype performed the analyses. Statistical analysis was performed as indicated; using one-way ANOVA or two-way ANOVA followed by posthoc testing of selected means with Bonferroni Multiple Comparison Test. A P-value of <0.05 was considered statistically significant. These results are reported as the mean +/− standard error of the mean (SEM) across the experiments. Statistical analysis and graphs were prepared using GraphPad Prism.

## Results

### CoupTFI Expression is not Downregulated in Foxc1-mutants

We previously identified a role for meningeal derived RA signaling in regulating the transition from symmetric proliferative divisions to asymmetric neurogenic divisions using an allelic series of Foxc1-mutants: Foxc1-hypomorphs (Foxc1^hith/hith^: Foxc1^H/H^), Foxc1-hypomorph-null hybrids (Foxc1^hith/lacZ^: Foxc1^H/L^) and Foxc1-nulls (Foxc1^lacZ/lacZ^: Foxc1^L/L^) [5]. Because CoupTFI overexpression in the cortex increased RA signaling [17] and there is evidence that RA signaling may promote expression of CoupTFI and CoupTFII in other systems [28], [29], we wondered whether dysregulated expression of CoupTFI in Foxc1-mutants might underlie aspects of the Foxc1 mutant phenotype.

**Figure 2:**
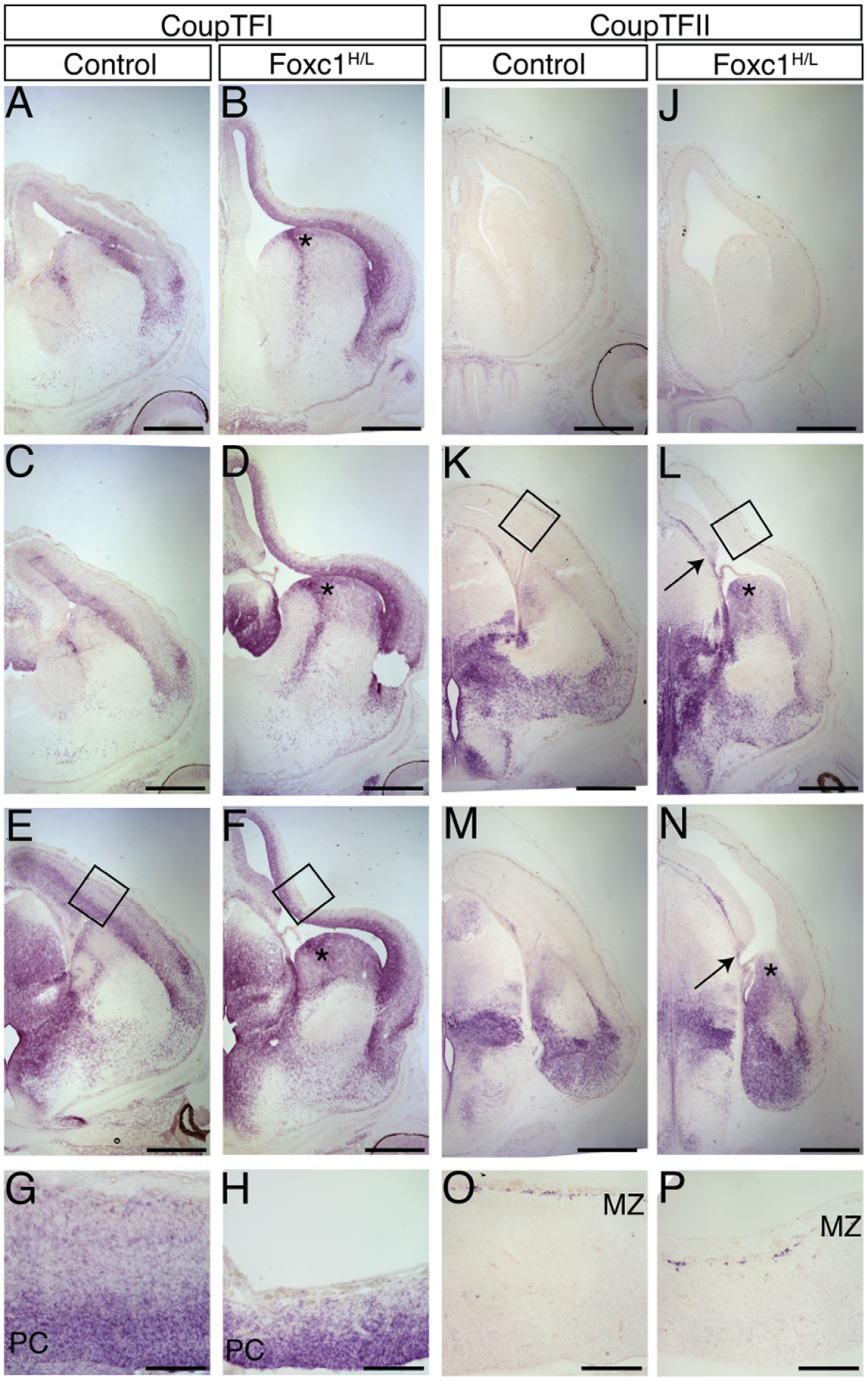
CoupTFI And CoupTFII Expression Is Not Downregulated Or Misexpressed In The Cortex Of Foxc1-Mutants At E14.5. *In situ* hybridization of coronal sections of control (A, C, E, G, I, K, M, O) and Foxc1^H/L^ (B, D, F, H, J, L, N, P) at E14.5 with CoupTFI probe (A–H) and CoupTFII probe (I–P). Higher magnification panels in E, F, K, L correspond to boxed regions in G, H, O, P. Scale bar: 500 µm in AF, I–N, 100 µm in G, H, O, P. Arrow in L, N points to the cortical hem. * in D, F, L, N indicates the ventral progenitor population. MZ: marginal zone.

At E14.5 CoupTFI is expressed in a high-ventral to low-dorsal pattern in cortical progenitor cells in controls (Figure 2A, C, E). In Foxc1-mutants, the overall pattern of CoupTFI expression in cortical progenitor cells was similar, although seemingly slightly upregulated (Figure 2B, D, F, H), whereas in ventral telencephalon progenitor cells CoupTFI expression was clearly upregulated (*, Figure 2B, D, F). In controls, expression of the related family member CoupTFII is largely restricted to the ventral forebrain and to Cajal Retzius cells in the cortical marginal zone (Figure 2I, K, M, O) [30]. In Foxc1-mutants CoupTFII expression was also upregulated in progenitor cells of the ventral telencephalon (*, Figure 2L, N) and cortical hem (arrow, Figure 2L, N). Thus, downregulation of either CoupTFI or CoupTFII expression in the cortex does not explain the neocortical phenotype of Foxc1-mutants.

### CoupTFI is Required for RA Rescue of Foxc1-mutants

Given the evidence that CoupTFI may be a mediator of RA signaling [14], [15] we wondered whether CoupTFI might be a required component of RA signaling in cortical progenitor cells. To address this we generated CoupTFI^−/−^;Foxc1^H/L^ compound mutants, and using our established model [5] examined the ability of dietary RA from E10 to E14.5 to rescue cortical development. We reasoned that if CoupTFI is a component of the RA signaling pathway, that RA might no longer rescue the Foxc1-mutants in the absence of CoupTFI.

**Figure 3:**
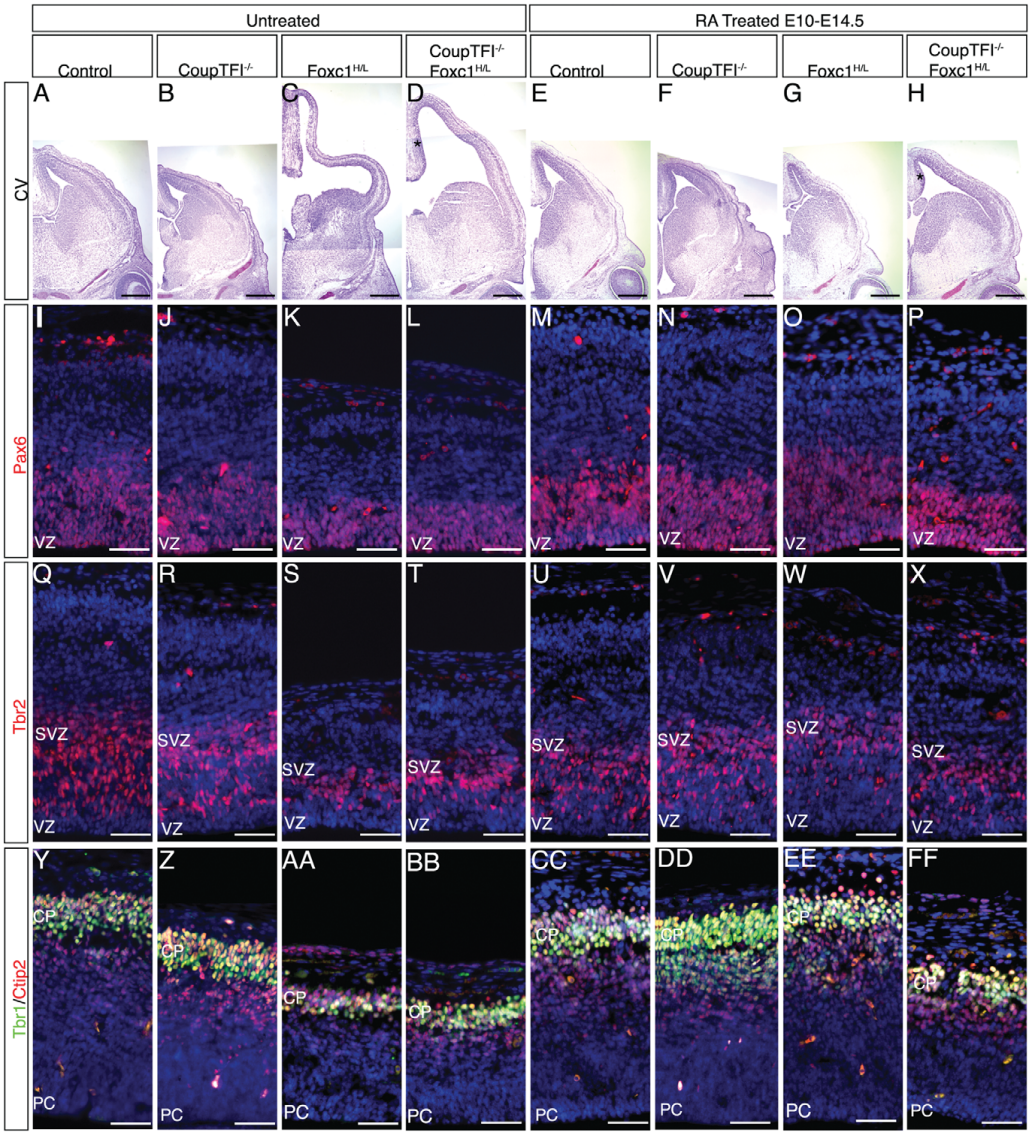
CoupTFI Is Required For The RA Mediated Rescue Of Foxc1-Mutants Animals. Cresyl Violet staining of E14.5 coronal forebrain sections of untreated and RA treated (E10–E14.5) embryos (A–H). Asterisk in D, H signifies the medial wall. Pax6 (red) immunohistochemistry of the dorsal cortex at E14.5 of untreated and RA treated (E10–E14.5 embryos) (I–P). Tbr2 (red) immunohistochemistry of the dorsal cortex at E14.5 of untreated and RA treated (E10–E14.5) embryos (Q–X). Tbr1 (green) and Ctip2 (red) immunohistochemistry of the dorsal cortex at E14.5 of untreated and RA treated (E10–E14.5) embryos (Y–FF). Sections are counterstained with DAPI (blue) (I–FF). Scale bar: 500 µm in A–H, 50 µm in I–FF. VZ: ventricular zone; SVZ: subventricular zone; PC: progenitor cell; CP: cortical plate.

**Figure 4:**
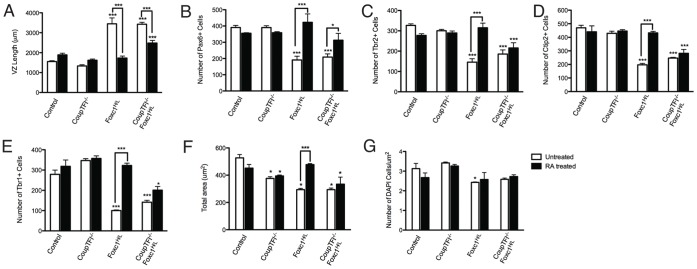
CoupTFI Is Required For The RA Mediated Rescue Of Foxc1-Mutants Animals. Quantification of dorsal forebrain length at E14.5 (A), Pax6 cell number at E14.5 (B), Tbr2 cell number at E14.5 (C), Ctip2 cell number at E14.5 (D), Tbr1 cell number at E14.5 (E), area of the counting window (F), density of DAPI^+^ cells in the counting window (G). Error bars represent SEM. A–G were analyzed by two way ANOVA: A: genotype (F_(3,25)_ = 55.86, p<0.001), treatment (F_(1,25)_ = 27.84, p<0.001), interaction (F_(3,25)_ = 49.98, p<0.001); B: genotype (F_(3,26)_ = 9.5, p<0.001), treatment (F_(1,26)_ = 48.66, p<0.01), interaction (F_(3,26)_ = 11.67, p<0.001); C: genotype (F_(3,27)_ = 19.23, p<0.001), treatment (F_(1,27)_ = 8.71, p<0.01), interaction (F_(3,27)_ = 14.55, p<0.001); D: genotype (F_(3,26)_ = 40.83, p<0.001), treatment (F_(1,26)_ = 18.45, p<0.001), interaction (F_(3,26)_ = 14.75, p<0.001); E: genotype (F_(3,26)_ = 47.99, p<0.001), treatment (F_(1,26)_ = 48.66, p<0.001), interaction (F_(3,26)_ = 14.91, p<0.001); F: genotype (F_(3,24)_ = 24.77, p<0.001), treatment (F_(1,24)_ = 8.158, p<0.01), interaction (F_(3,24)_ = 13.38, p<0.001); G: genotype (F_(3,25) = _10.33, p<0.001), treatment (F_(1,25) = _0.53, p = 0.47), interaction (F_(3,25)_ = 1.62, p = 0.2). *p<0.05, ***p<0.001 and indicate significance for Bonferroni’s Multiple Comparison Test posthoc analysis. Asterisks directly above the bar indicate significance from untreated control; within group differences are indicated by connected lines.

Previous studies identified that continued symmetric expansion of the radial glia population at the expense of asymmetric neurogenic divisions results in elongation of the cortical ventricular zone [5], [31]. We measured the length of the ventricular zone, from the pallial-subpallial boundary to the dorsal boundary of the cortical hem, in cresyl violet stained coronal sections at E14.5. In embryos that were not exposed to dietary RA the cortical ventricular zone length was elongated in both Foxc1^H/L^ and CoupTFI^−/−^;Foxc1^H/L^ mutants compared to controls (Figure 3C, D, Figure 4A). As previously reported, *in utero* RA treatment restored the ventricular zone length to untreated control levels in Foxc1^H/L^ mutants (Figure 3G, Figure 4A). On the other hand, in CoupTFI^−/−^;Foxc1^H/L^ mutants, RA treatment failed to fully rescue the phenotype to untreated control levels (Figure 3H, Figure 4A). The modest rescue observed in the CoupTFI^−/−^;Foxc1^H/L^ mutants (Figure 3D, H, Figure 4A), appeared to be due to shortening of the dorsomedial wall (*, Figure 3D, H).

Next, to determine whether CoupTFI is required for RA-mediated rescue of cortical progenitor cell properties in Foxc1^H/L^ mutants, we examined expression of radial glia (Pax6) and intermediate progenitor cell (Tbr2) markers at E14.5. While RA rescued the numbers of Pax6+ cells in both Foxc1-mutants and CoupTFI^−/−^;Foxc1^H/L^ animals (Figure 3I–P, Figure 4B), RA treatment did not restore the intermediate progenitor cell population to untreated control levels in CoupTFI^−/−^;Foxc1^H/L^ mutants (Figure 3Q–X, Figure 4C).

Finally, to investigate whether CoupTFI is also involved in the RA-mediated rescue of neuron production, we assayed early born neuron production by examining expression of Ctip2 and Tbr1 at E14.5 (Figure 3Y–FF, Figure 4D, E). While RA treatment restored neuronal differentiation to untreated control levels in Foxc1-mutants (Figure 3AA, EE, Figure 4D, E), RA treatment no longer rescued neuron production CoupTFI^−/−^;Foxc1^H/L^ double mutants (Figure 3BB, FF, Figure 4D, E).

The height of the 150 µm counting region differs across genotypes. We assessed the area of the counting region and the density of cells in this area to determine whether proportional changes in cell numbers account for the observed phenotypes (Figure 4F, G). The total area of the counting region was decreased in untreated and RA-treated CoupTFI mutants, untreated Foxc1-mutants, and untreated and RA-treated CoupTFI^−/−^;Foxc1^H/L^ animals (Figure 4F). RA-treatment restored the area of the counting region to untreated control levels in Foxc1^H/L^ animals, but not in CoupTF1^−/−^;Foxc1^H/L^ animals (Figure 4F). RA-treatment did not alter the decreased area of the counting window observed in CoupTFI^−/−^ animals (Figure 4F). Total cell density (DAPI quantification) within the counting region was slightly decreased in untreated Foxc1-mutants (Figure 4G). These findings suggest that changes in cell density do not account for the observed alterations in Foxc1-mutant mice. The cortical phenotype observed in Foxc1-mutants reflects alterations in the radial complexity of the cortex and are rescued by RA-treatment.

**Figure 5:**
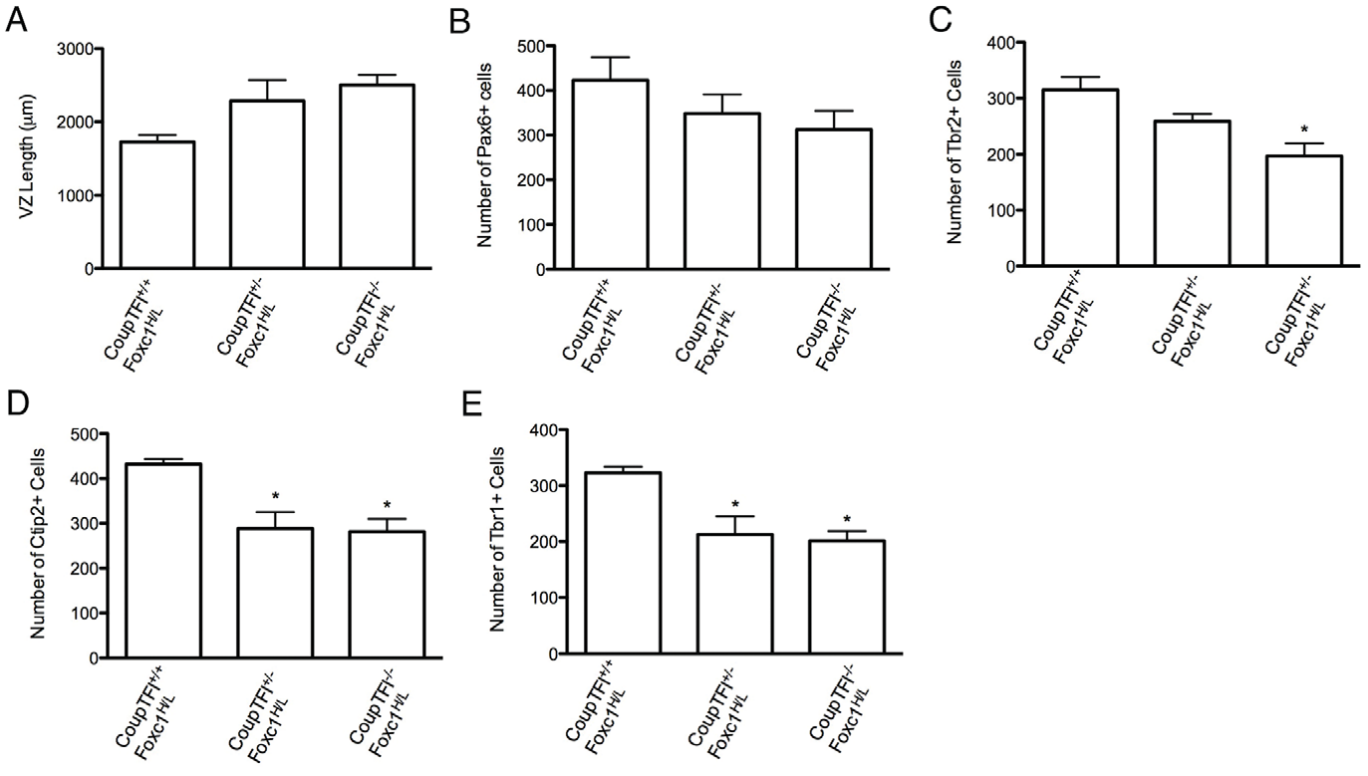
Dose response of CoupTFI in the RA-mediated rescue of Foxc1-mutants. Quantification of dorsal forebrain length at E14.5 (A), Pax6 cell number at E14.5 (B), Tbr2 cell number at E14.5 (C), Ctip2 cell number at E14.5 (D), and Tbr1 cell number at E14.5 (E) in RA treated animals. Note: the CoupTFI^+/+^;Foxc1^H/L^ and CoupTFI^−/−^;Foxc1^H/L^ data are the same as in Figure 4, but are being compared to CoupTFI^+/−^;Foxc1^H/L^ for this analysis. A–E were analyzed by one-way ANOVA: A: F_(2,13)_ = 3.7, p = 0.06; B: F_(2,12)_ = 1.3, p = 0.3; C: F_(2,10)_ = 8.5, p<0.01; D: F_(2,10)_ = 7.1, p<0.05; E: F_(2,10)_ = 6.9, p<0.05. *p<0.05 and indicates significance for Bonferroni’s Multiple Comparison Test posthoc analysis. Asterisks directly above the bar indicate significance from CoupTFI^+/+^Foxc1^H/L^ group.

We next examined the effect of CoupTFI dose in the RA-mediated rescue of the Foxc1-mutant phenotype, using RA-treated CoupTFI^+/+^;Foxc1^H/L^ (Foxc1^H/L^-mutants), CoupTFI^+/−^;Foxc1^H/L^, and CoupTFI^−/−^;Foxc1^H/L^-mutants. No significant difference in VZ length or Pax6 cell number was observed across the genotypes (Figure 5A, B). Tbr2 cell number was decreased in CoupTFI^−/−^;Foxc1^H/L^ but not in CoupTFI^+/−^;Foxc1^H/L^ animals (Figure 5C). Tbr1 and Ctip2 neuron number was significantly decreased in both CoupTFI^+/−^;Foxc1^H/L^ and CoupTFI^−/−^Foxc1^H/L^ animals (Figure 5D, E). These findings identify a CoupTFI dose response effect, with respect to the Tbr2^+^ population, however, the RA mediated production of Ctip2 and Tbr1 neuron number is affected by loss of even one copy of CoupTFI.

Taken together these studies indicate that CoupTFI is required for RA mediated rescue of many features of the Foxc1-mutant cortical phenotype. This supports our hypothesis that CoupTFI is a component of RA signaling during cortical development. However, because RA-treatment still rescued some of the CoupTFI^−/−^;Foxc1^H/L^ phenotype (i.e. numbers of Pax6^+^ radial glia cells and partial rescue of ventricular zone length), it seems likely that RA signaling in the cortex proceeds through other signaling interactions as well.

### Overexpression of CoupTFI in Cortical Progenitor Cells Partially Rescues Foxc1-mutants

Since CoupTFI is necessary for RA rescue of Foxc1 mutants (Figure 3, 4), we reasoned that overexpression of CoupTFI might be sufficient to rescue Foxc1-mutants without RA treatment. One possibility is that in the normal cortex activation of RAR/RXR by RA releases CoupTFI to function independently, allowing CoupTFI to interact with other targets. In this case, increasing the dosage of CoupTFI might allow this signaling to occur, even in the presence of low levels of RA that remain in the Foxc1-mutant cortex [5].

To increase CoupTFI dosage, we used mice that express CoupTFI in the cortical ventricular zone under the control of the D6-promoter element (D6-CoupTFI); this increased CoupTF1 expression ∼4-fold in cortical progenitor cells [17]. We generated D6-CoupTFI;Foxc1^H/L^ compound mutants, to assess whether increased CoupTFI dosage could rescue Foxc1^H/L^ cortical phenotypes independent of a RA treatment.

**Figure 6:**
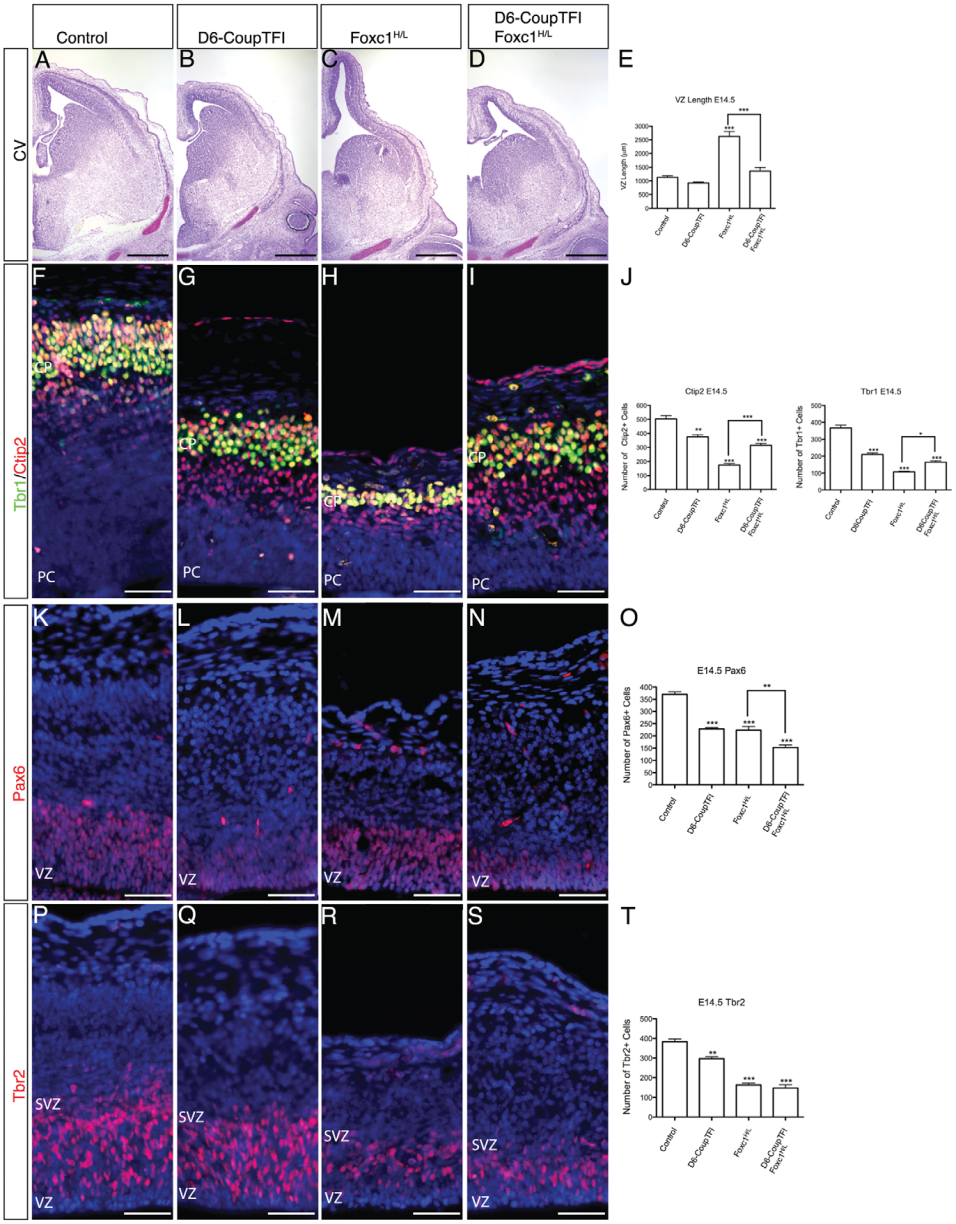
Overexpression Of CoupTFI In Cortical Progenitor Cells Partially Rescues The Cortical Phenotype Of Foxc1-Mutants. Cresyl Violet staining of E14.5 coronal forebrain sections (A–D). Quantification of dorsal forebrain length at E14.5 (E). Tbr1 (green) and Ctip2 (red) immunohistochemistry of the dorsal cortex at E14.5 (F–I). Quantification of Ctip2 and Tbr1 cell number at E14.5 (J). Pax6 (red) immunohistochemistry of the dorsal cortex at E14.5 (K–N) Quantification of Pax6 cell number at E14.5 (O). Tbr2 (red) immunohistochemistry of the dorsal cortex at E14.5 (P–S). Quantification of Tbr2 cell number at E14.5 (T). Sections are counterstained with DAPI (blue) (F–I, K–N, P–S). Scale bar: 500 µm in A–D; 50 µm in F–I, K–N, P–S. E, J, O, P were analyzed by one way ANOVA: E: F_(3,12)_ = 40.87, p<0.001; J: F_(3,12)_ = 62.28, p<0.001; F_(3,12)_ = 48.8, p<0.001; O: F_(3,12)_ = 74.38, p<0.001; P: F_(3,11)_ = 76.71, p<0.001. *p<0.05, **p<0.01; ***p<0.001 and indicate significance for Bonferroni’s Multiple Comparison Test posthoc analysis. Asterisks directly above the bar indicate significance from untreated control; within group differences are indicated by connected lines.

We measured the length of the ventricular zone at E14.5 (Figure 6A–E). One-way ANOVA revealed significant differences in ventricular zone length between genotypes (Figure 6E). Post-hoc testing revealed that the ventricular zone length was increased in Foxc1-mutants (Figure 6C, E), as previously described [5]. Overexpression of CoupTFI in dorsal cortical progenitor cells caused a subtle reduction in cortical length (Figure 6B, E). Strikingly, cortical length was restored to control levels in the D6-CoupTFI;Foxc1^H/L^ compound mutants (Figure 6D, E), providing strong evidence that CoupTFI overexpression is sufficient to restore the balance of asymmetric neurogenic divisions in Foxc1-mutants.

We next tested whether CoupTFI overexpression could rescue the deficit in early neurogenesis observed in Foxc1^H/L^ mice [5]. To do this we examined expression of Ctip2 and Tbr1 (Figure 6F–J). D6-CoupTFI mutants showed modest decrease in Ctip2 and Tbr1 positive cells (Figure 6G, J), probably due to an early depletion of the progenitor cell population [17]. Foxc1-mutants had a severe reduction of Ctip2 and Tbr1 neurons (Figure 6H, J). Despite the fact that both single mutants reduced neurogenesis, overexpression of CoupTFI in the D6-CoupTFI;Foxc1^H/L^ compound mutants increased the production of early born neurons compared to Foxc1^H/L^ mutants, although neuron numbers were not restored to control levels (Figure 6I, J).

While increased CoupTFI dosage increased neurogenesis in Foxc1^H/L^ mutant, it did not fully restore cortical neurogenesis. To explore the basis for the partial rescue, we examined the size of the cortical progenitor cell pools at E14.5, by examining markers of radial glia (Pax6) and intermediate progenitor cells (Tbr2). In both D6-CoupTFI and Foxc1-mutants the number of Pax6 cells was decreased (Figure 6L, M, O) [note: Pax6 expression was consistently weaker in D6-CoupTFI animals, Figure 6L, N and [17]]. However, the D6-CoupTFI;Foxc1^H/L^ compound mutants had a further decrease in the Pax6^+^ radial glia population (Figure 6N, O).

Tbr2^+^ intermediate progenitor cells were slightly decreased in the D6-CoupTFI mutants (Figure 6Q, T), whereas they were reduced by greater than 2-fold in the Foxc1-mutants (Figure 6R, T). Unlike the radial glia cells, there was no further reduction of intermediate progenitor cells in the D6-CoupTFI;Foxc1^H/L^ compound mutants (Figure 6S, T).

**Figure 7:**
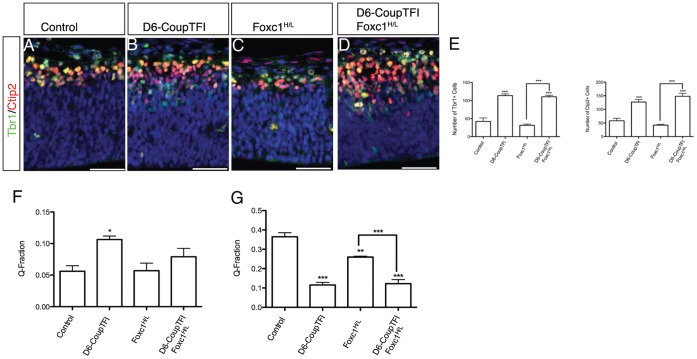
Overexpression Of CoupTFI In Cortical Progenitor Cells Increases Early Neuorgenesis. Tbr1 (green) and Ctip2 (red) immunohistochemistry of the dorsal cortex at E12.5 (A–D). Sections are counterstained with DAPI (blue). Quantificaiton of Ctip2 and Tbr1 cell number at E12.5 (E). Quantification of the Q-fraction at E12.5 (F) and E14.5 (G). E–G were analyzed by one way ANOVA: E: Ctip2 F_(3,11)_ = 35.5, p<0.001; Tbr1: F_(3,12)_ = 66.1, p<0.001; F: F_(3,12)_ = 5.2, p<0.001; G: F_(3,12)_ = 54.6, p<0.001. *p<0.05, **p<0.01; ***p<0.001 and indicate significance for Bonferroni’s Multiple Comparison Test posthoc analysis. Asterisks directly above the bar indicate significance from untreated control; within group differences are indicated by connected lines.

To assess the cellular basis of the partial rescue of the Foxc1-mutant phenotype we examined neuronal cell number at an earlier time point. D6-CoupTFI and D6-CoupTFI;Foxc1^H/L^ animals showed an increased production of both Ctip2 and Tbr1 cells at E12.5, compared to controls and Foxc1^H/L^ animals (Figure 7A–E). We also examined the rate of neuronal differentiation by examining the proportion of cells exiting the cell cycle at E12.5 (BrdU injected E11.5) and E14.5 (BrdU injected E13.5), termed the Q-fraction. At E12.5 an increased rate of cells exiting the cell cycle was observed in D6-CoupTFI embryos, although no significant difference in the Q-fraction was observed in D6-CoupTFI;Foxc1^H/L^ embryos (Figure 7F). By E14.5, the Q-fraction was decreased in D6-CoupTFI, Foxc1^H/L^, and D6-CoupTFI;Foxc1^H/L^ embryos compared to controls (Figure 7G), likely due to the depleted progenitor populations present at this age.

Early in neural development D6-CoupTFI promotes neurogenesis, increasing the number of neurons in D6-CoupTFI;Foxc1^H/L^ embryos. By E14.5, D6-CoupTFI;Foxc1^H/L^ compound mutants showed a reduction of Pax6^+^ radial glia progenitors greater than the single mutants, suggesting that CoupTFI overexpression is able to partially restore cortical growth and neurogenesis in Foxc1-mutants through reducing symmetric proliferative divisions, while maintaining asymmetric neurogenic divisions.

## Discussion

The meninges were traditionally considered to be a protective covering of the CNS, with roles limited to cerebrospinal fluid and blood circulation. In recent years several important additional signaling roles of the meninges have been established. The meninges produce Cxcl12, which guides neuronal migration [24], [32], [33], [34] and BMPs, which regulate the formation of the corpus callosum [35]. The meninges also synthesize RA, which regulates the onset of asymmetric divisions in the developing cortical neuroepithelium [5]. During development cortical neurons are born in a gradient from ventral to dorsal and rostral to caudal. Meningeal fibroblasts, derived from the neural crest, migrate in a ventral to dorsal wave from E10.5 and surround the developing forebrain and begin expressing RA synthesizing enzymes beginning at E12 [5], [36]. The migration of the meningeal fibroblasts and the initiation of expression of the RA synthesizing enzymes are coincident with the transition from symmetric proliferative divisions to asymmetric neurogenic divisions across the neurogenic gradient. The meninges are positioned and synthesize RA in a manner to temporally and spatially influence the regulation of radial glia, including controlling cortical neurogenesis and previous studies from our laboratory using Foxc1-mutant animals support this hypothesis [5]. This study supports the hypothesis that meningeal mediated RA signaling in radial glia involves the transcription factor CoupTFI.

The nuclear orphan receptor CoupTFI is a known modulator of retinoid responses in other systems [14], [15], CoupTFI regulates neuronal differentiation [17], [19], and D6-CoupTFI mice display elevated cortical RA signaling [17]. Our studies demonstrate a requirement for CoupTFI to allow RA mediated rescue of neurogenesis in Foxc1-mutants. We also found that overexpression of CoupTFI partially rescues the cortical phenotypes in Foxc1-mutants. Thus, our studies provide evidence that that CoupTFI interacts with RA signaling in the developing cortex.

A partial rescue of the ventricular zone length and the numbers of Pax6^+^ radial glia cells was observed in CoupTFI^−/−^;Foxc1^H/L^ mutants following RA treatment, whereas, Tbr2 and neuron cell numbers were not rescued. This suggests that RA regulation of symmetric proliferation (i.e. the expansion of the radial glia population) requires other signaling interactions as well. However, the RA mediated regulation of the production of intermediate progenitor cells and neurons, which proceed via asymmetric divisions, is dependent on CoupTFI. Consistent with this hypothesis, our overexpression studies (Figure 6), in which ventricular zone length and neurogenesis are rescued but the number of Pax6^+^ radial glia is further decreased, suggest that CoupTFI is sufficient to partially restore neurogenesis in Foxc1-mutants by promoting asymmetric neurogenic divisions at the expense of symmetric proliferative divisions. The cortical phenotype of Foxc1-mutants is evident from E12.5 and, based on expression of RA-synthesizing enzymes, meningeal RA synthesis initiates at around E12 [5]. In the D6-CoupTFI rescue model, CoupTFI is overexpressed in cortical progenitor cells from E10.5 [17], [27]. The partial rescue observed in D6-CoupTFI;Foxc1^H/L^ animals may also reflect a timing issue of expression of CoupTFI under the control of the D6-promoter and the early depletion of progenitor cells in D6-CoupTFI mice.

Abnormalities in the ventral forebrain are observed in Foxc1-mutant animals. The lateral ganglionic eminence (LGE) and medial ganglionic eminence (MGE) frequently appear smoother than controls, without a visible LGE/MGE sulcus, as visible in Figures 3 and 6. Our preliminary data suggest that the LGE/MGE are specified and patterned (unpublished observations, S Harrison-Uy and S Pleasure). The structural alterations are not rescued with RA treatment or overexpression of CoupTFI in cortical progenitor cells, which suggests that this component of the Foxc-1 mutant phenotype is not RA-dependent or dependent on structural changes in the cortex. In Foxc1-mutants the meninges covering the ventral forebrain is intact and RA-synthesizing enzymes are present [5], suggesting that these changes may be independent of RA signaling and may be dependent upon a signaling pathway that is directly regulated by Foxc1.

CoupTFI interacts with the retinoid receptors, RAR and RXR family members, altering the targets of CoupTFI and RA signaling [14], [15]. Our studies indicate that CoupTFI is necessary for the RA mediated rescue of Foxc1-mutants and is sufficient to rescue, in part, the cortical phenotype of Foxc1-mutants. Our results are consistent with a model where meningeal RA signaling alters the interaction of CoupTFI and RAR/RXR in cortical progenitor cells. This would allow CoupTFI and RAR/RXR to complex with additional signaling partners and enables the modification of the transcriptional targets of CoupTFI and RAR/RXR. We hypothesize that this signaling mechanism is an important regulatory switch necessary for cortical neurogenesis.

A number of important signaling pathways control cortical development including Fgf, Wnt, BMP, and Notch [37], [38]. Studies of CoupTFI mutants identified changes in the Fgf regulated pathways, Mapk/Erk kinase and PI3 Kinase/Akt, and Wnt signaling [17]. In the developing spinal cord Fgf signaling opposes RA signaling, regulating neural patterning and differentiation [39] and RA signaling is known to interact with Wnt signaling [40], [41], [42]. BMP and Notch signaling did not appear to be altered in the developing cortex of CoupTFI mutants [17], although CoupTFI has been implicated in Notch signaling during hair cell differentiation [43] and BMP signaling in bone development [44]. Interestingly, retinoic acid signaling has recently been shown to repress BMP signaling [45] and the retinol binding protein CRBP1 is a direct target of Notch1 [46]. Thus, CoupTFI and RA signaling may function to coordinate some of the major signaling pathways that control cortical development.
