# Correction: CoupTFI Interacts with Retinoic Acid Signaling during Cortical Development

**DOI:** 10.1371/annotation/f9bd989c-250a-49e9-b8e3-40aa929127d6

**Published:** 2013-08-23

**Authors:** Susan J. Harrison-Uy, Julie A. Siegenthaler, Andrea Faedo, John L. R. Rubenstein, Samuel J. Pleasure

Multiple funding organizations and grants were incorrectly omitted from the Funding Statement.

The Funding Statement should read: "This work was supported by the National Institute of Neurological Disorders and Stroke (K99-NS070920 to JAS; R01 NS34661 to JLRR); National Alliance for Research on Schizophrenia and Depression (AF); Nina Ireland (JLRR); Weston Havens Foundation (JLRR), and National Institute on Drug Abuse (R01 DA017627 to SJP). The funders had no role in study design, data collection and analysis, decision to publish, or preparation of the manuscript." 

